# A High-Performance Flag-Type Triboelectric Nanogenerator for Scavenging Wind Energy toward Self-Powered IoTs

**DOI:** 10.3390/ma15103696

**Published:** 2022-05-21

**Authors:** Yongjiu Zou, Minzheng Sun, Fei Yan, Taili Du, Ziyue Xi, Fangming Li, Chuanqing Zhu, Hao Wang, Junhao Zhao, Peiting Sun, Minyi Xu

**Affiliations:** 1 Dalian Key Lab of Marine Micro/Nano Energy and Self-Powered Systems, Marine Engineering College, Dalian Maritime University, Dalian 116026, China; zouyj0421@dlmu.edu.cn (Y.Z.); zheng3034304299@163.com (M.S.); yf1169@dlmu.edu.cn (F.Y.); dutaili@dlmu.edu.cn (T.D.); yyds@dlmu.edu.cn (Z.X.); lifangming@dlmu.edu.cn (F.L.); zcq@dlmu.edu.cn (C.Z.); hao8901@dlmu.edu.cn (H.W.); 2 Collaborative Innovation Research Institute of Autonomous Ship, Dalian Maritime University, Dalian 116026, China

**Keywords:** triboelectric nanogenerators, wind energy, distributed sensors, Internet of Things

## Abstract

Pervasive and continuous energy solutions are highly desired in the era of the Internet of Things for powering wide-range distributed devices/sensors. Wind energy has been widely regarded as an ideal energy source for distributed devices/sensors due to the advantages of being sustainable and renewable. Herein, we propose a high-performance flag-type triboelectric nanogenerator (HF-TENG) to efficiently harvest widely distributed and highly available wind energy. The HF-TENG is composed of one piece of polytetrafluoroethylene (PTFE) membrane and two carbon-coated polyethylene terephthalate (PET) membranes with their edges sealed up. Two ingenious internal-structure designs significantly improve the output performance. One is to place the supporting sponge strips between the PTFE and the carbon electrodes, and the other is to divide the PTFE into multiple pieces to obtain a multi-degree of freedom. Both methods can improve the degree of contact and separation between the two triboelectric materials while working. When the pair number of supporting sponge strips is two and the degree of freedom is five, the maximum voltage and current of HF-TENG can reach 78 V and 7.5 μA, respectively, which are both four times that of the untreated flag-type TENG. Additionally, the HF-TENG was demonstrated to power the LEDs, capacitors, and temperature sensors. The reported HF-TENG significantly promotes the utilization of the ambient wind energy and sheds some light on providing a pervasive and sustainable energy solution to the distributed devices/sensors in the era of the Internet of Things.

## 1. Introduction

The advancement of the Internet of Things era has greatly promoted the updating of a large number of low-power wireless sensors and wireless transmission equipment [1,2,3,4]. The number of distributed devices/sensors is enormous, reaching a figure in the billions, and they are widely disseminated. This quantity and scope mean greater energy demand, which will be doubled by 2035 and even grow to 1 GW per day by 2050 [5,6]. However, due to the limitation of natural resources and environmental pollution caused by energy production, a sustainable green energy is needed as a long-term reliable solution to meet our growing energy needs [7,8,9,10]. As a promising source of self-powered equipment, wind energy is widely distributed and highly available, especially in coastal areas, and is a promising renewable energy used in wireless sensors and wireless transmission equipment [11,12,13,14,15]. Traditional large-scale wind turbines can effectively convert wind energy into electricity by rotating blades driven by wind, but they are mainly used for large-scale grid power supply, and the later maintenance is not very convenient [16,17]. Over the past few decades, a variety of energy technologies, such as electromagnetic effect, piezoelectric effect, and triboelectric effect, have been scientifically applied to convert wind energy from the environment into electricity. However, due to the complex structure, large size, high initial investment, and inconvenient maintenance, electromagnetic wind energy harvesters are not appropriate in the harsh coastal environment [18,19]. In addition, piezoelectric wind energy harvesters are not suitable for application scenarios that require long-term operation at high wind speeds due to their poor flexibility and insufficient durability [20,21].

Wang’s team invented the triboelectric nanogenerator, in 2012, based on a combination of triboelectrification and electrostatic induction [22,23,24,25,26]. It has been proven to harvest fluttering wind energy with a wide frequency range due to its advantages of low cost, light weight, simple structure, stable output, wide choice of materials, and strong environmental adaptability [27,28,29,30,31,32,33]. Recently, many scholars have devoted themselves to the research of fluttering wind energy harvesters. Zhao et al. [34] first proposed a woven triboelectric nanogenerator flag (WTENG flag) with nickel-strip electrodes. When it flutters in the wind, the alternating contact and separation between the Kapton film and nickel strip can generate electricity, which can be used to harvest wind energy in any direction. However, due to the rigid metal electrode and extreme sensitivity to ambient humidity, the use of most of the demonstrated TENGs in environments with low wind speed and high humidity is limited [35,36,37]. Wang et al. [38] reported a humidity-resistant, wind-direction-adapting flag-type TENG capable of collecting wind energy in the environment and serving as a self-powered wind-speed and wind-direction sensor. On the basis of the above, Zhao et al. [39] modified the surface morphology of PTFE by sandpaper-grinding to improve the output performance, and systematically studied the effect of horizontal and vertical array arrangement on power output. However, the output performance of the single flag-type TENG needs to be improved. Since the gap between the two triboelectric materials is small and their bending degree is large and different when the flag-type TENG flutters under the action of the wind, it cannot be fully contacted and separated in each cycle, which severely limits the output of the flag-type TENG performance improvements. Therefore, in order to improve the output performance, it is urgent to find a new method to improve the degree of contact and separation between the triboelectric materials of the flag-type TENG.

**Figure 1:**
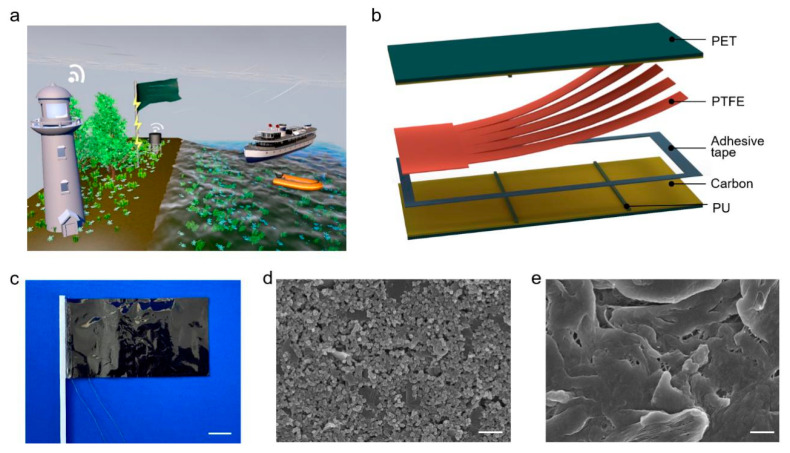
Structural design of the high-performance flag-type triboelectric nanogenerator (HF-TENG). (**a**) Application scenario of the HF-TENG. (**b**) Schematic illustration of the proposed HF-TENG. (**c**) Photograph of the as-fabricated flexible HF-TENG. Scale bar: 2 cm. (**d**) SEM image of the conductive-carbon electrode. Scale bar: 5 µm. (**e**) SEM image of the sandpaper-ground PTFE membrane. Scale bar: 5 µm.

We proposed a high-performance flag-type triboelectric nanogenerator (HF-TENG) for efficiently harvesting widely distributed and highly available wind energy. The HF-TENG, composed of two kinds of flexible membranes, PTFE and carbon-coated PET, could convert the kinetic energy of fluttering movement into electrical energy to power a large number of distributed devices/sensors, especially arranged in harsh coastal environments (Figure 1a). The supporting sponge strips were added between the two kinds of triboelectric materials, and the PTFE was divided into several pieces to obtain a multi-degree of freedom; this procedure improved the contact-and-separation degree of the two triboelectric materials and increased the output performance. When the pair number of supporting sponge strips is two and the degree of freedom is five, the maximum voltage and current achieved by HF-TENG are 78 V and 7.5 μA, respectively, which are both four times that of the untreated flag-type TENG. Moreover, the HF-TENG can be employed as a sustainable power supply for LEDs, capacitors, and temperature sensors. The proposed HF-TENG provides a sustainable green energy solution for distributed devices/sensors in the era of the Internet of Things.

## 2. Materials and Methods

**Fabrication of the HF-TENG**: Two surfaces of PTFE membrane with a length of 130 mm, a width of 60 mm, and a thickness of 30 μm were ground by sandpaper with different meshes. Two PET membranes with a length of 140 mm, a width of 70 mm, and a thickness of 25 μm were placed on the upper and lower surfaces of the PTFE membrane, and a conductive-carbon ink electrode with a micrometer thickness was uniformly coated on both PET membrane surfaces facing the PTFE. The 3M200C dual adhesive tape with a width of 5 mm and a thickness of 30 μm was used to bond the PTFE membrane with upper and lower PET membranes around the edge. Therefore, the gap distance between the conductive electrode and PTFE membrane is nearly the thickness of the dual adhesive tape. The flag surface and flag pole of HF-TENG are connected by adhesive tape. When the wind blows along the HF-TENG, it can flutter periodically and adjust to the wind direction, resulting in alternating voltage and current signals.

**Electrical Measurement.** The experiments for the HF-TENG are carried out in a wind tunnel with a dimension of 1.0 m (length) × 0.25 m (width) × 0.25 m (height). The wind speed varies from 5.0 m/s to 11 m/s. The blower is installed at the left end of the wind tunnel. An inverter is utilized to control the wind speed by adapting the rotating speed of the blower. The data acquisition and preprocessing system was built based on LabVIEW software and Ni acquisition card, which can realize real-time data acquisition and obvious rough analysis. The output signals of voltage, current, and transferred charge were measured by an electrometer (Keithley 6514, Keithley Instruments, Inc., Cleveland, OH, USA).

## 3. Results and Discussion

As schematically shown in Figure 1b, the flexible sandwich-like structure of the HF-TENG is composed of two carbon-coated PET membranes with the thickness of 25 μm and one piece of PTFE membrane with the thickness of 30 μm pasted together by adhesive tape. Here, PET is chosen as the substrate, while two layers of carbon membranes act as both a triboelectric material and a conductive electrode. The PTFE is divided into multiple pieces. Two layers of conductive carbon and PTFE form a free-standing contact-mode triboelectric nanogenerator. There are two pairs of supporting sponge strips (Polyurethane, PU) symmetrically attached to the conductive-carbon electrodes along the width. Figure 1c shows a photograph of the as-fabricated flexible HF-TENG. The detailed fabrication method of the HF-TENG is described in the Section 2. With a scanning electron microscope (SEM), the surface morphology of the conductive-carbon electrode and the sandpaper-ground PTFE membrane are obtained, as shown in Figure 1d and Figure 1e, respectively.

**Figure 2:**
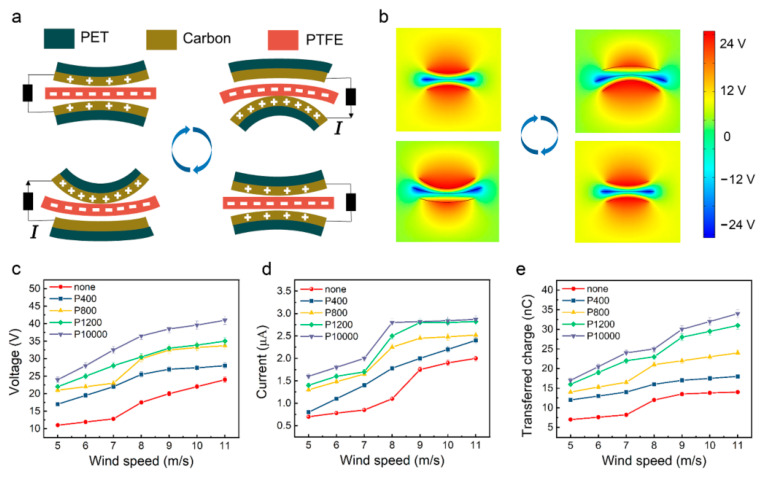
Working principle and grinding-improved output performance of the HF-TENG. (**a**) The working principle of the HF-TENG and the charge distributed in different stages. (**b**) The corresponding potential distribution calculated by COMSOL in a two-dimensional plane. (**c**) *V_OC_*, (**d**) *Isc*, and (**e**) *Qsc* of the HF-TENG under various wind speeds when the PTFE surface is ground with sandpaper of different mesh sizes.

The experimental apparatus and flutter characteristics of the flag-type TENG have been carried out by Wang et al. [38]; they approved the use of the flag-type TENG to harvest wind energy even under high humidity condition. Due to the different internal molecular structure, PTFE membrane and carbon-coated PET membrane can both generate flutter deformation under the influence of the wind, resulting in different bending degrees, which leads to the alternating contact and separation of PTFE and carbon electrodes. The specific working principle of the HF-TENG is demonstrated in Figure 2a. At the original state, two conductive electrodes and the PTFE membrane are separated, and there is no charge transfer. When the HF-TENG is bent upward by the wind, the PTFE makes contact with the lower conductive electrode. Due to the different electron affinity, the PTFE membrane is negatively charged, and the conductive electrode is positively charged. When they are separated, electrons will flow from the upper electrode to the lower one through the external circuit to balance the potential difference between them, generating a transient current. When the HF-TENG is bent downward by the wind, the PTFE contacts with the upper conductive electrode, which is positively charged. When the PTFE membrane is separated from the upper electrode, the electrons will flow in the opposite direction, and an opposite current will be generated in the external circuit. Therefore, alternating the contact and separation of the PTFE membrane and two conductive electrodes under the influence of wind will generate a current between the two conductive electrodes. The charge distribution and potential distribution of the HF-TENG are simulated via COMSOL Multiphysics software as shown in Figure 2b. Apparently, the simulation results are consistent with the above analysis.

According to the principle of the freestanding contact-mode TENG, the governing equation for HF-TENG can be written as follows:

(1) $$V=-\frac{Q}{C}+V_{OC}=-\left(\frac{d_{0}+g}{\varepsilon_{0}nA}\right)Q+\frac{2\sigma x(t)}{\varepsilon_{0}}$$

where *Q* is the amount of transferred charge, *V_OC_* is the open circuit voltage, *C* is the capacitance of the HF-TENG, *d*_0_ represents the effective thickness of the membrane, *ε*_0_ represents the relative dielectric constant of the air, *g* represents the distance between two electrodes, *n* is the divided pieces of PTFE (degree of freedom), *A* represents the actual contact area of each PTFE piece and the conductive-carbon membrane, *σ* is the surface charge density of the PTFE membrane and the conductive-carbon membrane, and *x*(*t*) denotes the distance between the PTFE membrane and the conductive-carbon membrane.

To systematically demonstrate the output performance of the HF-TENG, the mesh number of sandpaper, the pair number of supporting sponge strips, and the number of degrees of freedom are considered to be the most important factors in analyzing and comparing output performance. Experiments on the electrical output performance of the HF-TENG are carried out in an open-loop low-speed wind tunnel. The schematic of the experimental setup is illustrated in Figure S1. The *V_OC_*, *Isc*, and *Qsc* of the untreated flag-type TENG all increase with the increase in wind speed (Figure S2). To begin with, five HF-TENGs with the same size (length = 140 mm, breadth = 70 mm) are fabricated, and four of whose PTFE surfaces are ground with sandpaper of different mesh sizes to optimize the surface morphology and increase the effective contact area between the PTFE and the conductive-electrode membranes. Figure 2c shows the *V_OC_* of HF-TENG under various wind speeds when the PTFE surface is ground with sandpaper of different mesh sizes. From the experimental results, it can be found that as wind speed increases from 5 m/s to 11 m/s, the *V_OC_* of the untreated HF-TENG increases from 11 V to 23 V. Moreover, the output voltage increases with the increase in the mesh number of the sandpaper, and the P10000-ground HF-TENG obtained the optimal result. As the wind speed increases from 5 m/s to 11 m/s, the *V_OC_* of the P10000-ground HF-TENG increases from 24 V to 40 V. Similarly, the *Isc* and *Qsc* vary according to wind speed and the mesh number of the sandpaper in the same way as *V_OC_*, as shown in Figure 2d,e. However, the increase in the *Isc* and *Qsc* of the P10000-ground HF-TENG is not very obvious, so the following experiments utilize the sandpaper of P1200 for grinding.

**Figure 3:**
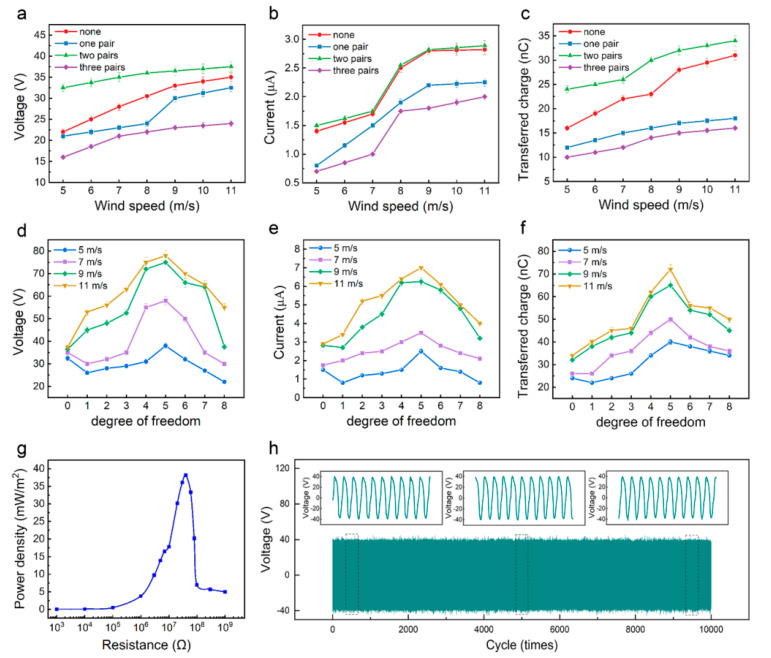
Electrical output performance of the HF-TENG under different pair numbers of supporting sponge strips and degrees of freedom. (**a**) *V_OC_*, (**b**) *Isc*, and (**c**) *Qsc* of the HF-TENG under various wind speeds when the pair number of supporting sponge strips is 0, 1, 2, 3. (**d**) *V_OC_*, (**e**) *Isc*, and (**f**) *Qsc* of the HF-TENG with different degrees of freedom. (**g**) Dependence of the peak power density on the loading resistances of the HF-TENG. (**h**) Electrical output stability of the HF-TENG.

Due to the small gap and large bending angle between the PTFE membrane and the conductive electrodes when the HF-TENG flutters under the influence of wind, it may not be able to contact and completely separate in every cycle, which leads to a lower output performance of the HF-TENG than expected. To improve the contact degree of the triboelectric surface in the process of fluttering, supporting sponge strips were first added between the PTFE membrane and the conductive-electrode membrane in the width direction. Three P1200-ground HF-TENG were prepared and then one, two, and three pairs of supporting sponge strips were symmetrically placed between the PTFE membrane and the conductive-electrode membrane, respectively. The experimental results obtained are shown in Figure 3a. It can be found that the *V_OC_* of HF-TENG with supporting sponge strips increases with the increase in wind speed. Meanwhile, the *V_OC_* of the HF-TENG with one and three pairs of supporting sponge strips is smaller than that of the HF-TENG without supporting sponge strips, while the *V_OC_* of the HF-TENG with two pairs of supporting sponge strips is larger than that of the HF-TENG without supporting sponge strips. The reason may be that too few or too many supporting sponge strips will limit the full contact and separation of the PTFE membrane and the conductive electrodes, and the two pairs of supporting sponge strips can better adapt to the HF-TENG’s fluttering movement, making it easier to achieve effective contact and separation between the triboelectric materials. The *V_OC_* of the HF-TENG with two pairs of supporting sponge strips can reach 37 V. Figure 3b,c show the relationship between the *Isc* and *Qsc* with changes in wind speed and the pair number of the supporting sponge strips, respectively. The variation tendency of the *Qsc* is almost the same as that of the *V_OC_*, while the *Isc* of the HF-TENG with two pairs of supporting sponge strips is almost the same as that of the HF-TENG without supporting sponge strips.

Another way to improve the contact degree of triboelectric surfaces is to divide the PTFE membrane into a multi-degree of freedom with equal area in the length direction. Each piece can move freely and flexibly, and there is no space limitation between each other. To explore the optimal degree of freedom of the PTFE membrane, nine different degrees of freedom of P1200-ground HF-TENGs with two pairs of supporting sponge strips are fabricated, and their output performance is compared. Figure 3d shows the relationship between the *V_OC_* of the nine different HF-TENGs mentioned above and the number of degrees of freedom. With the increase in degree of freedom, the *V_OC_* values of HF-TENGs show a trend of first rising and then falling, and all reach the maximum value when the degree of freedom is 5. As the degree of freedom reaches above 5, the width of each piece of the PTFE becomes smaller, the weight becomes too light, and stiffness becomes too small, making the PTFE pieces interfere with each other when swinging in the wind. It can also not guarantee that each PTFE piece is only swinging in its own orbit, and the phenomenon of torsion and overlap may occur, which weakens the overall contact and separation between the PTFE and the conductive electrode, affecting the internal power generation effect (Supplementary Movie S1). When the degree of freedom is 5, the overall contact and separation effect of triboelectric materials is the best. When the wind speed is 11 m/s, the *V_OC_* of the HF-TENG with a degree of freedom of 5 can reach 78 V. The change trend of the *Isc* and *Qsc* is consistent with that of the voltage, and shows the best output performance when the degree of freedom is 5 under any wind speed (Figure 3e,f). The maximum the *Isc* can reach is 7.5 μA, when the wind speed is 11 m/s. The maximum voltage and current of the HF-TENG are both four times that of the untreated flag-type TENG.

To systematically investigate the power output of the HF-TENG for effectively converting ambient wind energy into electric energy, external resistors were employed as load resistances to compare and analyze the power output of the five-degree-of-freedom HF-TENG with two pairs of supporting sponge strips for obtaining the peak power density when the wind speed is 11 m/s. The voltages and currents measured at variable external-resistance values from 10^3^ Ω to 1 GΩ for the HF-TENG are shown in Figure S3. The voltage increases with the increasing resistance while the current exhibits the reverse trend, but both the voltage and current tend to saturate at both high and low ends of the resistance. The power density of the HF-TENG varies according to external resistance, as shown in Figure 3g. The power density first increases in the low-resistance phase and then decreases in the high resistance phase. The peak power density reaches 38 mW/m^2^ when the external resistance is 40 MΩ. Wind-driven TENGs are mainly divided into flag type and up-and-down flap type. It can be seen from Table S1 that our work shows good power generation performance, especially in the flag-type TENG. Additionally, the HF-TENG also exhibited outstanding stability even after 10,000 cycles (Figure 3h), which results from the good stability of carbon coating on the PET and the flexibility of PTFE.

**Figure 4:**
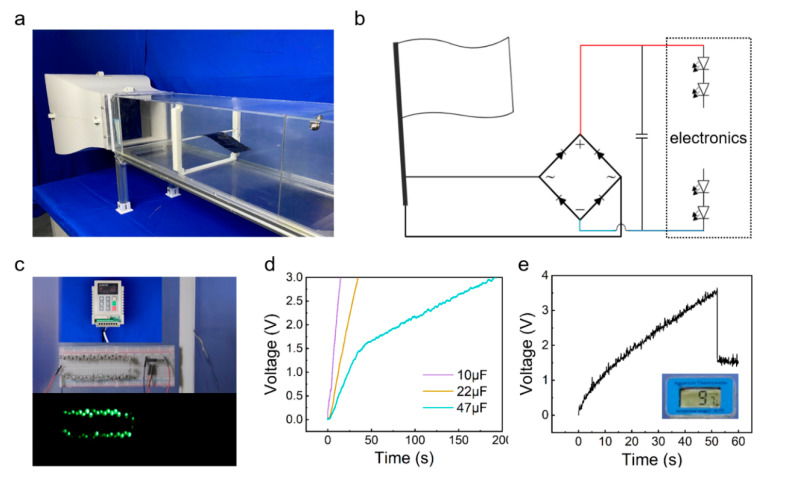
Demonstration of the HF-TENG as a sustainable power source. (**a**) Schematic of the experimental apparatus. (**b**) Proposed circuit diagram for continuously powering the electronics with the HF-TENG as a sustainable power source. (**c**) The charging curves of three different commercial capacitors (10 μF, 22 μF, and 47 μF) by the HF-TENG. (**d**) Demonstration of the HF-TENG to power a thermometer. (**e**) Demonstration of the HF-TENG to light up the LEDs.

The demonstrative experiments were carried out to exhibit the application of the HF-TENG. Figure 4a shows the actual scenario of the HF-TENG’s test experiment in an open-loop low-speed wind tunnel, where the HF-TENG is placed horizontally on a beam in the center of the wind tunnel. Figure 4b depicts the circuit diagram for continuously powering the electronics with the HF-TENG. The electronic signals generated by the HF-TENG are rectified by a bridge-rectifier circuit, then charged to a capacitor that acts as an energy-storage device, and finally the power is supplied to the electronics. Figure 4c shows a demonstration of the five-degree-of-freedom HF-TENG’s capacity to light up the LEDs. The video for lighting up the LEDs is shown in Supplementary Movie S2. To demonstrate the HF-TENG as a sustainable energy source, three different commercial capacitors (10 μF, 22 μF, and 47 μF) were continuously powered by the five-degree-of-freedom HF-TENG, as shown in Figure 4d. Figure 4e demonstrates that the thermometer is successfully lit up after charging the 22 μF capacitor for 50 s. The video for powering a thermometer is shown in Supplementary Movie S3.

## 4. Conclusions

A high-performance flag-type triboelectric nanogenerator was proposed for efficiently harvesting wind energy. By adding the supporting sponge strips between the two triboelectric materials and dividing the PTFE into several pieces to obtain a multi-degree of freedom, the contact-and-separation degree of the two triboelectric materials is improved and the output performance is increased. The maximum voltage and current achieved by the HF-TENG are 78 V and 7.5 μA, respectively, which are both four times that of the untreated flag-type TENG. The reported HF-TENG can power the LEDs, capacitors, and temperature sensors. Above all, the reported HF-TENG provides a new alternative for continuous power supply of distributed devices/sensors in the era of the Internet of Things.

## Supplementary Materials

The following supporting information can be downloaded at https://www.mdpi.com/article/10.3390/ma15103696/s1, Figure S1: The schematic illustration of the experimental setup; Figure S2: (a) *V_OC_*, (b) *Isc*, and (c) *Qsc* of the untreated flag TENG varied with wind speed; Figure S3: The voltages and currents measured at variable external resistance values from 10^3^ Ω to 1 GΩ for the HF-TENG. Table S1. A summary of structure and performance of various wind-driven TENGs. References [40,41,42,43,44,45,46,47] are cited in the supplementary materials. Movie S1: Slow-motion playback of multiple PTFE strips inside the 8-degree of freedom HF-TENG, Movie S2: Flag-type TENG lighting display, Movie S3: Flag-type TENG for powering thermometer.
