# Supplementary material for: A High-Performance Flag-Type Triboelectric Nanogenerator for Scavenging Wind Energy toward Self-Powered IoTs
Source: Materials (Basel). 2022 May 21;15(10):3696. doi: 10.3390/ma15103696 (PMC9143998; doi:10.3390/ma15103696)
Supplement: Supplementary file 1 [file materials-15-03696-s001.zip › Revised Supporting Information.pdf]

Supporting Information

A High Performance Flag-type Triboelectric Nanogenerator for Scavenging Energy Harvesting toward Self-powered IoTs

Yongjiu Zou [1,2,†], Minzheng Sun [1,†], Fei Yan [1,†], Taili Du [1,2], Ziyue Xi [1], Fangming Li [1], Chuanqing Zhu [1], Hao Wang [1], Junhao Zhao [1,*], Peiting Sun [1,2,*] and Minyi Xu [1,*]

1 Dalian Key Lab of Marine Micro/Nano Energy and Self-powered Systems, Marine Engineering College, Dalian Maritime University, Dalian, 116026, China; zouyj0421@dlmu.edu.cn (Y.Z.), zheng3034304299@163.com (M.S.), yf1169@dlmu.edu.cn (F.Y.), dutaili@dlmu.edu.cn (T.D.), yyds@dlmu.edu.cn (Z.X.), lifangming@dlmu.edu.cn (F.L.), zcq@dlmu.edu.cn (C.Z.), hao8901@dlmu.edu.cn (H.W.)
2 Collaborative Innovation Research Institute of Autonomous Ship, Dalian Maritime University, Dalian 116026, China; sunptg@dlmu.edu.cn

* Correspondence: haoger@dlmu.edu.cn.(J.Z.), sunptg@dlmu.edu.cn (P.S.), xuminyi@dlmu.edu.cn (M.X.)
† These authors contribute equally to this work.

Keywords: triboelectric nanogenerators, wind energy, distributed sensors, Internet of things

Supporting Figures and Tables:

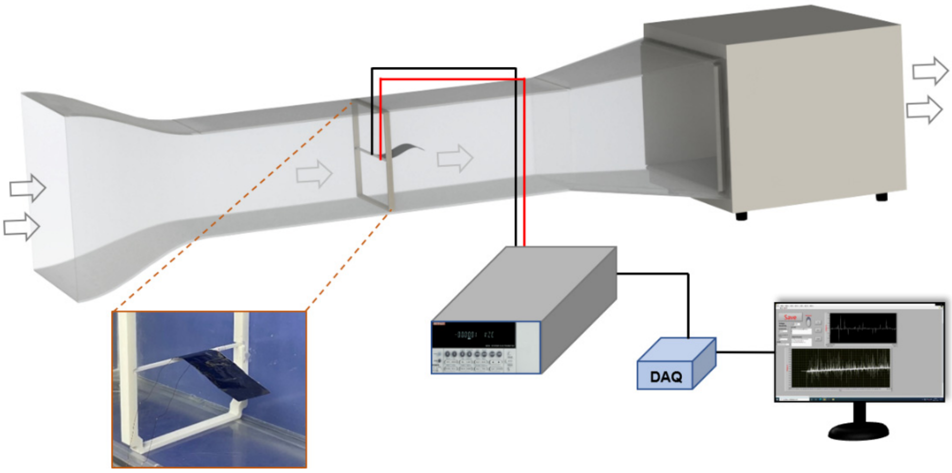

Figure S1. The schematic illustration of the experimental setup.

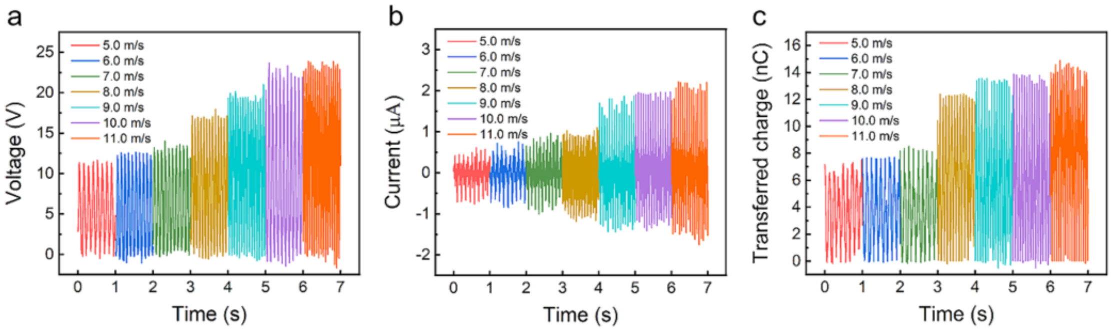

Figure S2. (a) *Voc*, (b) *Isc*, and (c) *Qsc* of the untreated flag TENG varied with wind speed.

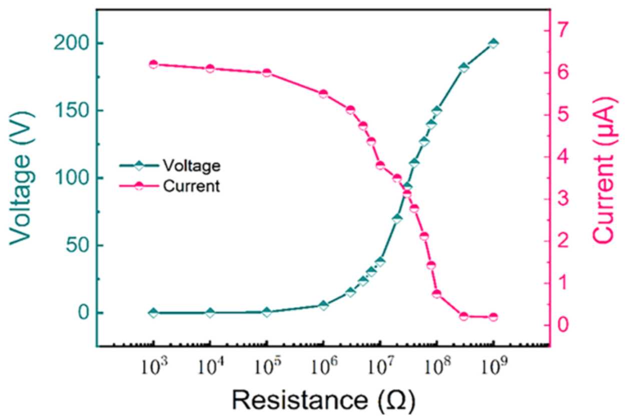

Figure S3. The voltages and currents measured at variable external resistance values from 10^3 Ω to 1 GΩ for the HF-TENG.

Table S1. A summary of structure and performance of various wind-driven TENGs

Device	Structure	Power density (mW/cm^3)	V (m/s)	Ref.
F-TENG	Flag type	0.0011	12.1	[1]
Flag-type TENG	Flag type	0.0408	7.5	[2]
FS-TENG	Flag type	0.049	6.96	[3]
F-TENG #3	Flag type	0.0598	7.0	[4]
Hybrid nanogenerator	Flap type	0.0616	10.2	[5]
Flow-driven TENG	Flag type	0.25	7.6	[6]
HF-TENG	Flag type	0.394	11	present
ATNG	Flap type	1	20	[7]
Wind-driven TENG	Flag type	2.2	18	[8]
Hybridized TENG	Flap type	2.46	15	[9]
Elasto-aerodynamics-driven TENG	Flap type	9	15	[10]

Reference

1. Su, Y.;Xie, G.;Xie, T.;Zhang, H.;Ye, Z.;Jing, Q.;Tai, H.;Du, X.;Jiang, Y., Wind energy harvesting and self-powered flow rate sensor enabled by contact electrification. *Journal of Physics D: Applied Physics* **2016**, *49*, 215601.

2. Wang, Y.;Yang, E.;Chen, T.;Wang, J.;Hu, Z.;Mi, J.;Pan, X.;Xu, M., A novel humidity resisting and wind direction adapting flag-type triboelectric nanogenerator for wind energy harvesting and speed sensing. *Nano Energy* **2020**, *78*, 105279.

3. Ye, C.;Dong, K.;An, J.;Yi, J.;Peng, X.;Ning, C.;Wang, Z. L., A triboelectric–electromagnetic hybrid nanogenerator with broadband working range for wind energy harvesting and a self-powered wind speed sensor. *ACS Energy Letters* **2021**, *6*, 1443-1452.

4. Zhao, Z.;Wei, B.;Wang, Y.;Huang, X.;Li, B.;Lin, F.;Ma, L.;Zhang, Q.;Zou, Y.;Yang, F.;Pang, H.;Xu, J.;Pan, X., An array of flag-type triboelectric nanogenerators for harvesting wind energy. *Nanomaterials (Basel)* **2022**, *12*, 721.

5. Ma, M.;Zhang, Z.;Liao, Q.;Zhang, G.;Gao, F.;Zhao, X.;Zhang, Q.;Xun, X.;Zhang, Z.;Zhang, Y., Integrated hybrid nanogenerator for gas energy recycle and purification. *Nano Energy* **2017**, *39*, 524-531.

6. Wang, S.;Mu, X.;Yang, Y.;Sun, C.;Gu, A. Y.;Wang, Z. L., Flow-driven triboelectric generator for directly powering a wireless sensor node. *Adv Mater* **2015**, *27*, 240-248.

7. Guo, H.;He, X.;Zhong, J.;Zhong, Q.;Leng, Q.;Hu, C.;Chen, J.;Tian, L.;Xi, Y.;Zhou, J., A nanogenerator for harvesting airflow energy and light energy. *J. Mater. Chem. A* **2014**, *2*, 2079-2087.

8. Zheng, H.;Zi, Y.;He, X.;Guo, H.;Lai, Y.-C.;Wang, J.;Zhang, S. L.;Wu, C.;Cheng, G.;Wang, Z. L., Concurrent harvesting of ambient energy by hybrid nanogenerators for wearable self-powered systems and active remote sensing. *Acs Applied Materials & Interfaces* **2018**, *10*, 14708-14715.

9. Wang, S.;Wang, X.;Wang, Z. L.;Yang, Y., Efficient scavenging of solar and wind energies in a smart city. *ACS Nano* **2016**, *10*, 5696-5700.

10. Wang, S.;Mu, X.;Wang, X.;Gu, A. Y.;Wang, Z. L.;Yang, Y., Elasto-aerodynamics-driven triboelectric nanogenerator for scavenging air-flow energy. *ACS Nano* **2015**, *9*, 9554-9563.
